# Chorea*Read before the Physicians’ Club, September 28, 1888.

**Published:** 1888-11

**Authors:** Irving M. Snow

**Affiliations:** 371 Porter Avenue; Buffalo, N. Y.


### *CHOREA.**

By IRVING M. SNOW, M. D., Buffalo, N. Y.

I am well aware that an enormous literature, at the disposal of any student, exists upon the subject of chorea, yet, within the last few years, diseases of the nervous system have been so thoroughly and successfully studied that many facts have come to light which explain some of the curious phenomena of the disease. My interest in chorea was awakened by the many cases which I saw in the Children's Hospital in London, by observing the numerous variations in the disease, and also by hearing the lectures of Dr. Gowers, whose discussion of any subject is most masterly, and whose powers of diagnosis and concise description are almost ideal. I shall only attempt to discuss the chorea of childhood, omitting the very interesting chorea major, choreiform movements of hysterical origin, and also the chorea of adults.

Although in a subject dead of chorea, changes are generally found in both nervous and circulatory systems; the lesions found in any one organ are not constant, and chorea is, therefore, classed with the fast-diminishing group of functional diseases. Neverthless, it is well to add that the severity of the motor disturbances, and the frequent grave complications, render it extremely probable that a profound change in the nutrition of the nerve elements takes place.

A physician is occasionally called to see a child, whose grimaces and peculiar movements have aroused the anxiety of its parents

* Read before the Physicians' Club, September 28, 1888.

and teachers. They relate that, after a fright or fall, or often without apparent cause, the child commences to jerk and twitch; and so characteristic and plain are the patient's symptoms, that the parents themselves have generally reached the conclusion that the child has St. Vitus's dance. The child is, indeed, in a ludicrous and pitiable condition. The face, when at rest, has a vacant and apathetic expression, but more often the forehead is wrinkled, the eyelids and corners of the mouth twitch, and if the physician be incautious enough to place his thermometer in the patient's mouth, he will probably see it bitten in two by the sudden and convulsive action of the jaws. The head is twitched to the right and left, and the child is generally unable to feed itself or walk, on account of its utter lack of control over its arms and legs.

In these violent motor disturbances we may distinguish (1) the element of irregular muscular spasm; (2) the element of incoördination; and (3) the element of paresis. It may be difficult, in the first stages of the malady, to distinguish the jerky, awkward movements of a choreic patient from the habitually restless and uneasy manner of a nervous child. Children, also, who are mentally deficient, show a curious lack of control over their hands and feet. However, the physician, by questioning, may soon discover if the motor disturbances be congenital, or if they affect muscles which have heretofore been intelligently used. In a typical case of chorea, all parts of the body participate in the so-called "muscular insanity." The child is literally unable to keep still, the arms are quickly flexed and extended, are rotated and twisted into every conceivable position, the face twitches, and the legs are no longer to be trusted to carry the body of the little patient.

*Incoördination.*—It will commonly be found that the child is unable to feed itself. If it attempt to carry a glass of water to the mouth, the tremulous hand makes so many excursions to the right and left that the glass is usually empty when it reaches the mouth; or, if the little patient be asked to walk, it goes in a zigzag direction, stumbling over little obstacles, the knees and ankles giving out at unexpected times.

*Paresis.*—Almost all choreic patients suffer from a loss of motor power. The amount of paresis varies from a scarcely perceptible weakness of grasp, to a condition in which the upper or lower extremities can neither be raised or moved. The paresis is almost always most marked in the upper extremities. In fact, Gowers asserts that when a child has a paralysis, in one arm, gradual in its development, the cause is invariably chorea. The truth of this statement is easily shown, for, if in such a case the sound arm be watched, the characteristic twitching may be observed, and the symptoms may be still further developed by causing the patient to hold the hand above the head. I saw a case of this kind in the Children's Hospital in London, in which the right arm was practically useless, the child using the left arm to pull itself into a sitting position. The paresis usually affects the muscles most strongly agitated. In the case just mentioned, however, the paresis appeared coincident with the development of general chorea. The association of spasm and paresis may be observed by causing the child to press your hand; the hand will be quickly and unevenly grasped, the pressure, however, almost instantly relaxing. The physician will further notice that any sustained muscular exertion is impossible; also, that any voluntary movement is executed suddenly, the patient hoping to accomplish its object before the incoördination overcomes the muscles.

A choreic child speaks with great difficulty and hesitation, the voice is broken, staccato; articulation of long words is impossible, the laryngoscopic image shows spasm and incoördination of the laryngeal muscles. Anything eaten is swallowed with a jerk. Thus the patient is harassed by a laryngeal and pharyngeal chorea. The ocular symptoms are often quite striking. Strabismus is found in one-third of the cases; nystagmus and diplopia are quite common. Even the respiratory muscles, especially the diaphragm, may participate in the neurosis, causing irregular respiration and sometimes cyanosis. Chorea of the heart, the French authors notwithstanding, is said not to exist; the irregularity of the pulse and apex-beat and impurity of the first sound are ascribed to the interrupted respiration and, perhaps, to impending endocarditis. The disease shows a slight predilection for the left side, the twitching commencing most frequently in the left arm, then passing to the left leg, then the left side of the face, and afterwards passing to the right side and becoming general. Or, the movements may appear on one side for a while, then completely subside, and afterward attack the other half of the body. Thus, the disease may remain unilateral throughout, invading both sides at different times. Pure hemichorea is occasionally met with, but is often more apparent than real. The movements are usually slight on one side and very violent on the other.

However, I remember the case of a young girl who was frightened by a sea-lion at a circus. She commenced to twitch on the left side going home, and afterward suffered from an attack of left hemichorea.

I tested the reflexes in some twenty cases of chorea in the Children's Hospital, and found a great variation—in some the right jerk was exaggerated; in some normal, and in others lost. The pupils are generally dilated and sluggish to light. Rosenthal has carefully tested the electrical reactions in cases of hemichorea. With the constant current the electrical excitability is greater on the affected than on the healthy side. This condition, however, is present only during the height of the disease, and disappears with the return to health.

*Sensory and Psychical Phenomena.*—Sensory disturbances in uncomplicated chorea are limited, I believe, to hyperesthesia; the anesthesia of the French authorities is present only in hysterical subjects. Psychical symptoms can be found, however, in almost every choreic child. Indeed, a change in the disposition is often one of the primary symptoms of the malady. The child becomes irritable, forgetful, lachrymose, is extremely emotional, and gives way to the most violent bursts of passion at anything which offends its childish fancy. Rilliet and Barthez mention hallucinations of sight and hearing as occurring frequently in chorea. The little patients see ghosts and death's heads around their beds; they believe fierce lions and tigers are about to devour them, and hear strange voices in the air. All of these delusions are of a sad and terrible nature; or, a delirium usually mild, but occasionally maniacal in character, may develop. Sometimes the mental state is one of depression, the children may pass into a state of dementia, lying apathetic and indifferent to surrounding objects. It is well to remember, however, that choreic children have habitually a vacant, unnatural expression, which is increased by the inability to articulate, and one must discriminate between the physiognomy of the disease and true mental hebetude All choreic symptoms are increased by excitement. Allan McLane Hamilton, however, believes that choreic movements are sometimes under the control of the will, while occasionally they are exaggerated by an effort to repress them. With such violent disturbances of mind and body, it is no wonder that in general chorea the little patients become thin and anemic. Sleep is interrupted, the stools are irregular, the skin becomes harsh and dry. In fact, the choreic spasm may become so violent and continuous that sleep is impossible. In these cases, insomnia and exhaustion form an exceedingly grave complication. Sex seems to exert an important influence on the severity of the disease. The mildest and the most violent cases occur in girls, while the chorea of boys is generally moderate in type. An elevation of temperature is present in about twelve per cent. of the cases.

*Intercurrent Diseases*—Other affections tend to complicate the malady, notably intercurrent endocarditis and rheumatism. If the heart of a choreic child be auscultated, a murmur, often systolic, will commonly be heard at the apex. Much dispute has arisen as to whether the cardiac murmur in chorea is organic or hemic. Before the expert diagnosticians of this club I will not venture to go minutely into the clinical distinctions. It is well, in order to ascertain if the murmur be soft or harsh, to auscultate over the jugular, the pulmonary and mitral orifices, and to trace out the lines of propagation of the murmur. When the physician has satisfied himself that organic heart disease really exists, he should endeavor to ascertain whether the lesion be old or recent. With hypertrophy or dilatation of the right ventricle and accentuation of the second pulmonic sound, indicating old mitral trouble, in all probability the endocarditis antedates the chorea. French observers find a murmur in every choreic child. Sinkler discovered a murmur which he believes to be often anemic in one-third of his cases. An official report of the British Medical Association shows that thirty-two per cent. of a large number of cases of chorea investigated were complicated with either primary or secondary endocarditis; and a further analysis shows that in one-half of the cases the endocarditis preceded the chorea, and in one-half it was an intercurrent malady.

Secondly, the little patient suffering with chorea may be attacked by acute articular symptoms, which may be coincident with the endocarditis, or may be entirely independent of it. In fact, articular rheumatism may be associated with chorea in three distinctions, viz.: (1) It may precede the neurosis; (2) it may appear as an intercurrent disease; (3) it may attack the patient months after the subsidence of all motor disturbances. Rheumatism in children is often slight and transitory in character. I have seen a case of acute endocarditis in which the only rheumatic manifestation was a stiff neck. A careful inquiry into the previous condition of a choreic child will often elicit a history of growing pains, stiff neck, muscular tenderness, or swollen and painful joints. Another manifestation of the rheumatic poison is often found in chorea. During the disease small subcutaneous lumps, called by Barlow rheumatic nodules, may appear upon the tendons and ligament. Their favorite seat is the scalp, spine, and joints. They appear and pass away with the disease, and are nearly always associated with endocarditis. In the Children's Hospital I saw a most remarkable case of this kind in a choreic child, with a distinctly rheumatic history. The nodules were of the size of a horse-chestnut, and were found above both ears on the occiput, along the spine, and on the ankles, knees and wrists. They were not tender, and passed away with the disease, which was of a mild type. English statistics show that twentyfive per cent. of children with chorea have suffered previously from acute articular rheumatism, and that twenty-two per cent. more have suffered from articular or cardiac disorders, or from vague rheumatic pains. Thus forty-seven per cent. of English choreic children have a rheumatic history. This proportion is greater than in the United States, for Osler, of Philadelphia, reports that only fifteen per cent. of his cases showed evidences of antecedent rheumatism. Other variations, probably due to climate, race, etc., are found in French and Austrian statistics.

Both intercurrent rheumatism and endocarditis usually pursue a mild course. Nevertheless, the endocarditis seems to bear some relation to the violence and persistence of the disease, and often leaves behind permanent valvular trouble. Both boys and girls are equally subject to the rheumatic complication. It must be remembered that while in adult life rheumatism is vastly more common in males, in childhood it attacks the sexes with equal impartiality.

The ordinary type of chorea is moderate in character. The severe motor and physical disturbances occur only in exceptional cases; intercurrent rheumatism and endocarditis run a mild course, and the disease almost invariably ends in complete recovery. The choreic movements attain their greatest violence at the end of two weeks, and then gradually subside. Chorea is almost invariably a sub-acute or chronic disease, lasting from four to twelve weeks. Hereditary weakness of constitution, unfavorable or noisy surroundings, and mental excitement, render a prognosis as to the duration of the malady exceedingly unwise. Rilliet and Barthez made an analysis of 235 cases of chorea resulting in a cure, twelve per cent. in one month or less; forty per cent. lasted from one to two months; twenty-five per cent. from two to three months, and the remaining twenty-three per cent. from three to twelve months.

More than forty per cent. of children with chorea suffer from a return of the disease. These relapses are apparently excited by most trivial causes—excitement, errors in diet, or slight exposures. They may appear weeks, or even months, after the subsidence of the first attack. A relapse is usually milder and shorter than a first attack, and affects girls more often than boys. With recurring chorea, heart disease plays an important role. Most patients who suffer from repeated attacks of chorea either have or acquire endocarditis. In all instances in which three attacks of chorea occurred, the patient had endocarditis and was of the female sex.

Chorea is found in all climates and seasons, but is more common in damp, cold weather. Morris Lewis, of Philadelphia, has recently published some tables showing that the number of storm centers passing over that city coincide with the prevalence of chorea.

The disease evidences an extraordinary predilection for the female sex, three-fourths of all cases being girls. With them, chorea is severe in character, more protracted in course, and more prone to relapses. The favorite age is between the period of the second dentition and puberty. Chorea under five years is very rare. During my attendance in the Children's Hospital I saw a case in a girl two years and eleven months old. The exciting cause was a fall, followed by convulsions and afterward by chorea. Between five and ten years the greatest number of first attacks take place; but, including relapses, Gowers saw the greatest number in the thirteenth year. Instances are reported where intercurrent disease, like pneumonia and scarlatina, have had the singular effect of lessening or even curing the choreiform movements.

Less than two per cent. of the cases of chorea die. Endocarditis, the exhaustion and insomnia following violent chorea, septicemia from contusion, or decubitus, are the most frequent causes of death. Of eighty fatal cases of chorea reported by Dr. Sturges, endocarditis with vegetations about the mitral valve were found in all but five. Exudations, hyperemia, with minute hemorrhages, have been found in all parts of the brain and spinal cord. Of especial interest are the emboli found lodged in the smaller vessels of the cerebral ganglia and cortex.

*Etiology.*—I have already alluded to the predisposing influences of age and sex. All causes which tend to enfeeble or unduly excite the nervous system play an important role in the production of chorea. The highly stimulating influences of city life, a hereditary tendency to epilepsy, insanity, or chorea itself, are to be regarded as predisposing to the disease. Allan McLane Hamilton asserts that twenty per cent. of the school children in New York City suffer from St. Vitus's dance. Whether he believes the uncertain, awkward movements of nervous children to be choreic in origin, I am unable to say. The annual examinations in the public schools are said to be a fruitful source of chorea. I cannot but think that the lack of physical exercise among the children in the city schools has much to do not only with the prevalence of chorea but also with the causation of headache, dyspepsia, neurasthenia, general feebleness of constitution and tendency to bronchitis. The children are confined in dark, badly ventilated rooms for from five to six hours daily, are stimulated and harassed by the systems of marking and by frequent examinations. During the short recess, which may be abbreviated or omitted by the caprice of the teacher, they stand listlessly about the school-rooms or walk aimlessly up and down the yard or sidewalk without the strength or inclination to play. If a system of regular gymnastics, such as is practiced in the schools in Berlin, were in force in America, city children would not go out into life handicapped, as they often are, by a feeble and neurotic constitution. The close association of the rheumatic diathesis with chorea is familiar to you all. Tapeworms are accused of exciting choreic symptoms. It is certain that the disease is sometimes speedily terminated after the evacuation of intestinal parasites. As to the production of chorea by imitation, in hospitals choreic children are placed side by side with children with rheumatism, endocarditis, and other maladies, without fear of the disease spreading through the wards. Nevertheless, it occasionally happens that the sight of a person in violent hysteria will produce chorea in a previously healthy subject; but emotion of any kind may cause chorea in a child predisposed to the disease.

Fright is the only directly exciting cause with which we are familiar. About twenty-five per cent. of all cases are produced by sudden mental shock. The patient may begin to twitch immediately, or after the lapse of some days. I remember attending a patient who was frightened by the cries of her mother, who was suffering from a cerebral disease; another who was terrified by the crowded traffic about the Bank of England. Curiously enough, a sudden mental shock, which produces chorea in children, may cause paralysis agitans in adults. Fright may cause chorea in children with heart disease; and in those who are perfectly sound, general or hemichorea is equally liable to occur.

*Pathology.*—The localization and pathology of all nervous diseases is most interesting, for our increasing knowledge of the physiology of the brain and spinal cord points out the intimate connection between symptoms and the functions of the nervous tracts; but, unfortunately, the pathology of chorea is still debatable ground, two theories being held by the profession, (1) the embolic theory, and (2) a blood poison.

The embolic theory of chorea is based upon the belief that chorea is dependent upon endocarditis, that minute vegetations are detached from the valves and are carried in the arteries, as emboli, to the brain. They are arrested by the diminishing calibre of the cerebral arteries, and plug up the minute vessels in the motor tracts in the cerebral peduncles and ganglia, and also in the motor areas around the fissure of Rolando. Here, by irritation or by other disturbances of nutrition, they excite the motor phenomena peculiar to chorea. This theory is strengthened by numerous autopsies, which almost invariably show recent endocarditis, and often cerebral emboli, by the frequent and undeniable connection between heart disease and chorea, by the fact that choreic symptoms may be present in subjects with chronic disease of the motor tracts, and, lastly, by the experiments of Angel Money, who, by injecting starch granules into the carotids of dogs, produced choreic symptoms, due, evidently, to the artificial emboli. Nevertheless, I believe that all of the manifestations of chorea are unexplained by the embolic theory, and will present for your consideration the objections to it, and also the theory of an altered blood state, an idea now widely accepted by the profession. The seat of chorea in man is evidently the brain, as demonstrated by the psychical disturbances, the involvement of the facial and other cerebral nerves, the spread of the motor symptoms from arm to leg, the cessation ot the movements during sleep, the brain being quiet and the cord more active in sleep. It is also certain that motor influences are arranged at the cortex, the most important elements in chorea being the incoördination and spasm.

In opposition to the embolic theory of chorea, it is urged (1) that fatal cases of chorea have been reported without endocarditis and cerebral embolism; (2) that the etiological value of endocarditis only holds good when it precedes the chorea. Now, as a matter of fact, cardiac disease may be present before the chorea, may appear during its course, or may follow, at an interval of weeks or months, the cessation of the choreic movements. Cases of chorea, moreover, occur purely from emotional causes in subjects without rheumatic history or symptom of endocarditis. Fright does not detach vegetations from a healthy valve, nor does muscular agitation lead to endocarditis.

But we cannot lightly explain away the close association between rheumatism, endocarditis, and chorea, and the old humeral theory has revived and is almost universally accepted. Rilliet and Barthez believe that chorea is a cerebral manifestation of rheumatism in children. Gowers and most of the profession hold that chorea is produced by an altered blood state, allied to, but not identical with, the condition present in rheumatism, which produces a transitory effect upon the motor areas, either upon the whole region or upon centers of single groups of matter, as in facial tic or in hemichorea.

*Treatment.*—As chorea generally terminates in complete recovery, it is often asserted that direct medication is of no use. As a matter of fact, if a choreic child be taken from school and put to bed, the choreiform movements will diminish in violence, and the disease will commonly run a moderate course, but many cases do not improve under this expectant treatment, and may be greatly helped by well-directed therapeusis. It is the almost universal testimony that arsenic is directly beneficial, especially with children who have not the favorable environments of rest and quiet. With cases in which the movements are so violent as to prevent sleep and rest, Fowler's solution, diluted with glycerine, given hypodermically, is of extreme value. Cases of obstinate chorea of months' duration often improve under the hypodermic use of arsenic. The objection to this plan is that the pain of the injection may lead to violent emotional attacks. In the absence of articular symptoms, anti-rheumatic remedies are of no use. Chloral often does great good in controlling the restlessness incident to the earlier stages, and inducing sleep. Strychnine is indicated if there be much paresis or muscular debility, and in chronic chorea iron is always valuable in the profound anemia which accompanies the disease. Sometimes spraying the spine with ether will quiet the movements. Dr. Barlow, of the Children's Hospital, was wont to emphasize the importance of keeping the bowels open and promoting the action of the skin by diaphoretics and by warm baths. He also advocated mild hydropathic treatment, douching the patient with lukewarm or cold water, the child being afterwards well rubbed and put to bed.

The last addition to the therapeutics of chorea comes from France. M. Legroux gives antipyrine in large doses—forty-five grains daily—to his cases, and reports astonishing success, curing his cases in from six to twenty-seven days. Chorea occasionally becomes so violent that the patient may throw himself against the bed, floor, etc., and the resulting injuries are exceedingly dangerous, numbers of patients dying from abscesses or septicemia. In these cases, it is well to secure the patient with bandages, or, better still, to lay him on a mattress on the floor.

Gymnastics have been highly extolled by M. Sée, but English and American authorities have not had the same success which M. Sée achieved. City children are greatly benefited by frequent drives, and obstinate cases will often very quickly convalesce when the little patient is shifted to some seaside resort.

371 Porter Avenue.

